# Characterization of KPC-82, a KPC-2 Variant Conferring Resistance to Ceftazidime-Avibactam in a Carbapenem-Nonsusceptible Clinical Isolate of Citrobacter koseri

**DOI:** 10.1128/AAC.00150-21

**Published:** 2021-06-17

**Authors:** Francois Lebreton, Brendan W. Corey, Christi L. McElheny, Alina Iovleva, Lan Preston, Katie R. Margulieux, Robert J. Cybulski, Patrick Mc Gann, Yohei Doi, Jason W. Bennett

**Affiliations:** a Multidrug-Resistant Organism Repository and Surveillance Network (MRSN), Walter Reed Army Institute of Research, Silver Spring, Maryland, USA; b Division of Infectious Diseases, University of Pittsburgh School of Medicine, Pittsburgh, Pennsylvania, USA; c Department of Pathology and Laboratory Services, Brooke Army Medical Center, Joint Base San Antonio-Fort Sam Houston, Texas, USA

**Keywords:** CRE, *Citrobacter koseri*, KPC, carbapenems, ceftazidime-avibactam

## Abstract

KPC-82 is a KPC-2 variant identified in a carbapenem-nonsusceptible Citrobacter koseri that confers high-level resistance to ceftazidime-avibactam. Genomic analysis revealed that *bla*_KPC-82_ is carried by a chromosomally integrated Tn*4401* transposon (disrupting porin gene *phoE*) and evolved by a 6-nucleotide tandem repeat duplication causing a two-amino-acid insertion (Ser-Asp) within the Ala_267_-Ser_275_ loop. Similar to related KPC variants, KPC-82 showed decreased carbapenemase activity when expressed in a heterologous background and remained susceptible to carbapenem/β-lactamase inhibitor combinations.

## INTRODUCTION

Carbapenem-resistant *Enterobacteriaceae* (CRE) are a significant threat to modern medicine. In particular, isolates producing carbapenem-hydrolyzing β-lactamase enzymes (carbapenemases) are increasingly prevalent and a cause for further concern given their ability to spread, the severity of infections, and the lack of effective therapeutics (1). Though colistin and tigecycline have been used as first-line treatment, newer antimicrobials with better safety profiles and potent activity against CRE are increasingly being employed as preferable therapeutic options (2).

Among them, ceftazidime-avibactam (CZA) is a β-lactam/β-lactamase inhibitor combination recently introduced into clinical practice (2). It has proven active against serine β-lactamases, including Klebsiella pneumoniae carbapenemases (KPC), which otherwise confer resistance to most β-lactams and β-lactam/β-lactamase inhibitor combinations (1). Despite limited clinical use worldwide, acquired resistance has been reported in multiple independent occurrences and by several mechanisms in both patients with or without a history of CZA therapy (3–10). Most frequently, resistance is caused by KPC variants exhibiting amino acid substitutions, insertions, or deletions in one of 4 loops (loop Leu_102_ to Ser_106_, Ω-loop Arg_164_ to Asp_179_, or loops Cys_238_ to Thr_243_ and Ala_267_ to Ser_275_) (11). At the time of writing (April 2021), 82 *bla*_KPC_ alleles have been deposited in GenBank, including 20 conferring CZA resistance. In this report, we use genomic and molecular genetic approaches to characterize KPC-82, a KPC-2 variant that confers CZA resistance.

**FIG 1:**
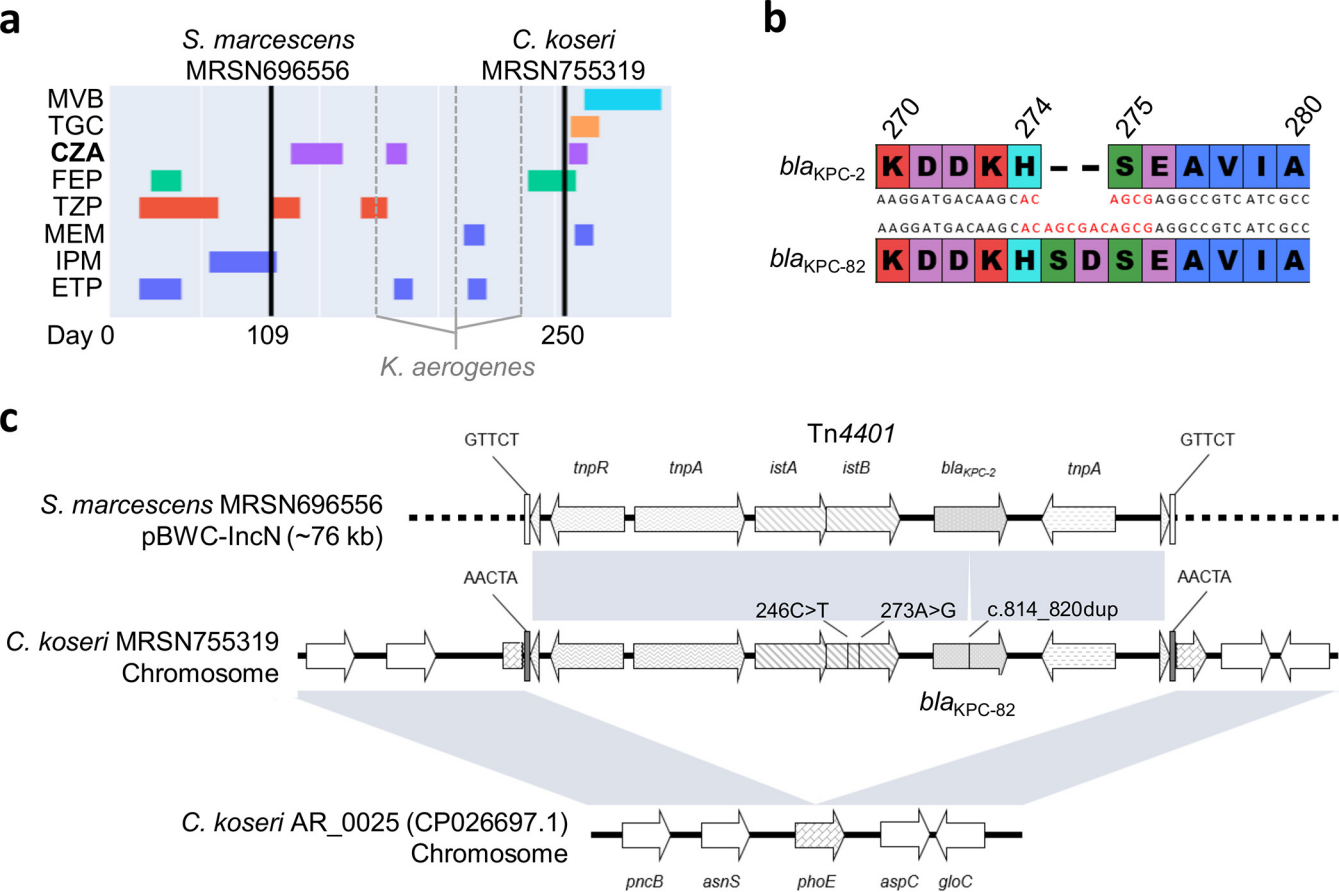
Identification and characterization of KPC-82. (a) Patient treatment course and sample collection. (b) Nucleotide and amino acid alignment (Ambler numbering and Clustal color scheme) of KPC-2 and KPC-82. (c) Alignment of the plasmid-borne KPC-2 carrying Tn*4401* (MRSN 696556) with the chromosomally integrated KPC-82 carrying Tn*4401* (MRSN 755319) and its corresponding insertion site in reference genome C. koseri AR_0025.

Citrobacter koseri MRSN 755319 was cultured from the blood of a patient in a U.S. hospital in 2020. The patient had been hospitalized for several months after suffering a gunshot wound to the abdomen. During this time, the patient had frequent infections caused by multidrug-resistant (MDR) bacteria, including a recurrent respiratory infection due to a carbapenem-susceptible Klebsiella aerogenes (days 159, 197, and 231) as well as a bloodstream infection caused by a carbapenem-resistant (CR), *bla*_KPC-2_-carrying Serratia marcescens (MRSN 696556, day 109), that ultimately resolved after ∼4 weeks of treatment with CZA (Fig. 1A). Two and a half months after CZA was discontinued, the patient developed another infection, and blood cultures yielded C. koseri (MRSN 755319, day 250). The isolate was carbapenem resistant (Table 1), and the *bla*_KPC_ gene was detected using the Cepheid Xpert Carba-R assay. On day 252, the patient was prescribed tigecycline and CZA, which was substituted on day 260 with meropenem-vaborbactam (MVB) following extended antibiotic susceptibility testing (AST) that indicated the isolate was nonsusceptible to CZA (MIC, 128 μg/ml) but susceptible to MVB (MIC, 0.125 μg/ml).

**TABLE 1:** MICs of β-lactams for isolates S. marcescens MRSN 696556, C. koseri MRSN 755319, and recombinant strains E. coli TOP10 with or without KPC-82 or KPC-2

β-lactam(s)^*a*^	MIC (μg/ml) of:				
	S. marcescens^*b*^ MRSN 696556 (KPC-2)	C. koseri^*b*^ MRSN 755319 (KPC-82)	E. coli^*c*^ TOP10 (KPC-82)	E. coli^*c*^ TOP10 (KPC-2)	E. coli^*c*^ TOP10 (pBCSK)
Ampicillin	>16	>16	>16	>16	≤8
Ampicillin-sublactam	>16	>16	>16	>16	≤4
Piperacillin-tazobactam	>64	>128	32	>128	≤8
Ceftriaxone	>64	>32	32	>32	≤0.5
Cefepime	>16	>32	16	32	≤4
Ceftazidime	16	>16	>16	>16	4
Ceftazidime-avibactam	8	128	64	0.5	0.25
Ceftolozane-tazobactam	ND^*d*^	>16	>16	>16	≤1
Aztreonam	ND	>16	>16	>16	≤1
Ertapenem	>8	4	0.5	4	≤0.25
Imipenem	>4	4	1	>8	≤0.5
Meropenem	>4	2	≤0.5	>8	≤0.5
Meropenem-vaborbactam	0.125	0.125	ND	ND	ND

a Tazobactam and avibactam were added at a fixed concentration of 4 μg/ml.

b Performed in duplicate using a Vitek 2 in the MRSN College of American Pathologists (CAP)-accredited laboratory.

c Performed in two biological duplicates (distinct transformants confirmed by Sanger sequencing) using a Gram-negative GN4F AST plate (Thermo Fisher).

d ND, not determined.

As part of routine surveillance of MDR organisms, isolates S. marcescens 696556 and C. koseri 755319 were forwarded to the Multidrug-Resistant Organism Repository and Surveillance Network (MRSN). Whole-genome sequencing was performed on an Illumina MiSeq sequencer (Illumina, Inc., San Diego, CA), and genomes were processed as previously described (12). For S. marcescens 696556, long-read sequencing was performed using a MinION sequencer (Oxford Nanopore Technologies). Base-calling was performed using Guppy (configuration r9.4.1_450bps_hac) and filtered using Filtlong (https://github.com/rrwick/Filtlong), and hybrid assembly was performed using Unicycler (13). 

Genome analysis revealed that CR and CZA-susceptible (Table 1) S. marcescens MRSN 696556 carried the *bla*_KPC-2_ allele. In contrast, CR and CZA-nonsusceptible C. koseri MRSN 755319 carried a mutated *bla*_KPC-2_ allele (hereby named *bla*_KPC-82_; GenBank accession no. MW485086) and no other acquired β-lactamase. The mutated allele was identical to *bla*_KPC-2_ with the exception of a 6-nucleotide (ACAGCG) tandem repeat (TR) insertion causing a two-amino-acid insertion (Ser-Asp) between positions 274 and 275 (Ambler numbering) in the KPC protein (Fig. 1B). TR insertions within the KPC Ala_267_ to Ser_275_ loop have been reported previously (11), including KPC-50, a KPC-3 variant with a three-amino-acid insertion (Glu-Ala-Val) at this exact position (3).

To investigate whether the two-amino-acid insertion identified within KPC-82 was responsible for the phenotypic resistance to CZA, the *bla*_KPC-82_ gene was cloned into vector pBCSK (Stratagene, La Jolla, CA) and expressed in E. coli TOP10. AST showed that KPC-82 conferred resistance to all β-lactams, including ceftazidime, as well as high-level resistance to CZA (Table 1). Importantly, and similar to KPC-50 (3), E. coli expressing *bla*_KPC-82_ remained susceptible to the carbapenems (ertapenem, imipenem, and meropenem).

Further investigations into the genetic context of *bla*_KPC-82_ in MRSN 755319 revealed that it was carried by an ∼10-kb Tn*4401*-like transposon that inserted into the chromosome and disrupted the gene coding for the outer membrane protein PhoE (Fig. 1C and D). Porin loss, such as OprD in P. aeruginosa (14) and OmpK36 in K. pneumoniae (15), has been widely implicated in β-lactam and carbapenem resistance in other bacterial species. Notably, PhoE downregulation has been hypothesized as a possible reason for carbapenem resistance in K. pneumoniae (16), suggesting that its inactivation in MRSN 755319 could cause the otherwise unexplained low-level carbapenem resistance (Table 1).

Interestingly, in S. marcescens MRSN 696556 from the same patient, the *bla*_KPC-2_ allele was also carried by a nearly identical Tn*4401* (only 2 synonymous mutations in *istB* in addition to the TR insertion in *bla*_KPC_). However, unlike MRSN 755319 but similar to previous reports (17), this transposon was not chromosomally located and was instead carried by an ∼76-kb IncN plasmid named pBWC01 (Fig. 1C and D). The backbone of pBWC01 was absent in MRSN 755319, but both S. marcescens and C. koseri isolates carried an identical ∼4-kb Col440i-type plasmid (Fig. 1C). Similar Col440i cryptic plasmids have been identified in a variety of *Enterobacteriaceae* and have been documented to coconjugate with a larger IncN KPC-carrying plasmid (including across genus, *in vitro*) (18). Altogether, and despite missing intermediate isolates, a hypothesis for the emergence of *bla*_KPC-82_ would be that (i) both plasmids cotransferred from *Serratia* to *Citrobacter* within the host, and (ii) Tn*4401* inserted into the chromosome of *Citrobacter* while the remaining of pBWC01 was lost. In this proposed chain of events, whether *bla*_KPC-82_ evolved from *bla*_KPC-2_ in *Serratia*, as a result of CZA exposure, or once acquired by *Citrobacter* MRSN 755319 still remains unresolved.

In summary, a novel KPC-type enzyme conferring resistance to CZA was identified from a multidrug-resistant C. koseri. Similar to other KPC mutants conferring resistance to CZA, KPC-82 showed decreased carbapenemase activity and remained susceptible to carbapenem/β-lactamase inhibitor combinations, including meropenem-vaborbactam, which successfully cleared the infection in this patient.

### Data availability.

Genomes of S. marcescens MRSN 696556 and C. koseri MRSN 755319 have been deposited at NCBI (BioProject accession no. PRJNA692233).
